# Neuroanatomical and neurocognitive correlates of delusion in Alzheimer’s disease and mild cognitive impairment

**DOI:** 10.1186/s12883-024-03568-5

**Published:** 2024-03-06

**Authors:** Seyul Kwak, Hairin Kim, Keun You Kim, Da Young Oh, Dasom Lee, Gieun Nam, Jun-Young Lee

**Affiliations:** 1 Department of Psychology, Pusan National University, Busan, Republic of Korea; 2 Department of Psychiatry, Seoul National University College of Medicine & SMG-SNU Boramae Medical Center, Seoul, 07061 Republic of Korea; 3 Department of Medical Device Development, Seoul National University College of Medicine, Seoul, Republic of Korea

**Keywords:** Alzheimer’s disease, Dementia, Cortical thickness, Delusion, Neuropsychiatric symptoms, Cognitive function, Neuropsychological test

## Abstract

**Background:**

Neuropsychiatric symptoms and delusions are highly prevalent among people with dementia. However, multiple roots of neurobiological bases and shared neural basis of delusion and cognitive function remain to be characterized. By utilizing a fine-grained multivariable approach, we investigated distinct neuroanatomical correlates of delusion symptoms across a large population of dementing illnesses.

**Methods:**

In this study, 750 older adults with mild cognitive impairment and Alzheimer’s disease completed brain structural imaging and neuropsychological assessment. We utilized principal component analysis followed by varimax rotation to identify the distinct multivariate correlates of cortical thinning patterns. Five of the cognitive domains were assessed whether the general cognitive abilities mediate the association between cortical thickness and delusion.

**Results:**

The result showed that distributed thickness patterns of temporal and ventral insular cortex (component 2), inferior and lateral prefrontal cortex (component 1), and somatosensory-visual cortex (component 5) showed negative correlations with delusions. Subsequent mediation analysis showed that component 1 and 2, which comprises inferior frontal, anterior insula, and superior temporal regional thickness accounted for delusion largely through lower cognitive functions. Specifically, executive control function assessed with the Trail Making Test mediated the relationship between two cortical thickness patterns and delusions.

**Discussion:**

Our findings suggest that multiple distinct subsets of brain regions underlie the delusions among older adults with cognitive impairment. Moreover, a neural loss may affect the occurrence of delusion in dementia largely due to impaired general cognitive abilities.

## Background

Late-life cognitive impairment and dementia are accompanied by profound burdens and distress due to loss of cognitive abilities and socioemotional changes. One of the devastating factors of caregivers’ burden comes from significant deviation in patients’ social behavior. Unfortunately, such neuropsychiatric symptoms (NPS) are ubiquitous in the neurological condition of dementia or cognitive impairment [1, 2]. Especially, psychotic symptoms of delusion characterized by paranoia and misidentification are highly prevalent in dementia of Alzheimer’s disease (AD) [3]. These delusional symptoms can manifest as falsely inferring that others will harm themselves, or misidentifying others as people different from the real world and acting inappropriately in the social context. Impaired social behavior and inappropriate judgments due to delusions lead to a greater burden in the patient’s care [4, 5].

There are, however, profound individual variabilities, in that only some proportion of dementia patients exhibit delusional changes while others remain intact in delusion symptoms. It remains largely unclear the exact nature of the gap between patients with or without such neuropsychiatric changes. Previous studies have explored whether observable neural differences account for the presence of delusion and were able to identify multiple brain regional correlates that might account for the symptoms [6–12]. While findings indicated the frontal lobe as a converging site, many other regions showed inconsistent positive findings [13, 14]. More troubling was that the comprehensive literature review pinpointed almost every anatomical lobe as a correlate of delusion, leaving a non-specific theoretical account for delusion symptoms [15]. A multitude of regional findings and coarse definitions of neuroanatomical measurements remain difficult in elucidating the neural bases of delusion in Alzheimer’s disease.

Another perspective in understanding delusion regards understanding the symptoms as a shared consequence of cognitive impairments that stably affect the manifestation of symptoms. Neurodegeneration can affect heteromodal brain structures that take charge of both social appropriateness and cognitive tasks [16, 17]. Degeneration in functional hub regions can lead to impairment in neurocognitive endophenotypes including attention, executive control, and verbal memory deficit, which can account for the underlying mechanism of psychosis [18]. Similarly, neuropsychological profiling has identified that verbal episodic memory and executive control task performance account for delusions in older adults with neurocognitive disorders [19, 20].

In combination with both neuroimaging measures and cognitive test performance, only scarce studies directly examined the extent to which cognitive functions share the neural bases of behavioral symptoms. In one previous study, the brain structural comparison between the delusion-positive and delusion-negative groups was largely attenuated when the group difference in cognitive function was additionally adjusted, implying a mediating role of the cognitive function in explaining the delusional symptom [21]. However, the results were examined as overall cognitive function and lacked information in specific domains of cognitive function that played the mediating role on delusion. Moreover, the brain regional measurements relied on broad anatomical definitions which hinder more valid testing of regions as homogeneous functions and covarying properties. Since a number of disparate and broad brain regions are reported as neural correlates of delusion in dementia, it remains difficult to advance core theories that explain the individual differences of delusion in dementia.

In the current study, we explored cortical thickness correlates of delusion symptoms among older adults with Mild Cognitive Impairment (MCI) and dementia of Alzheimer’s disease. We assessed more fine-grained cortical thickness definitions and utilized multivariate summary measures of regional patterns to identify correlates of delusions. Furthermore, the mediation model tested whether the brain structural bases of delusion are mediated by multiple domains of cognitive functions including executive function, processing speed, memory, visuospatial, and language domains.

## Methods

### Participants

**Fig. 1:**
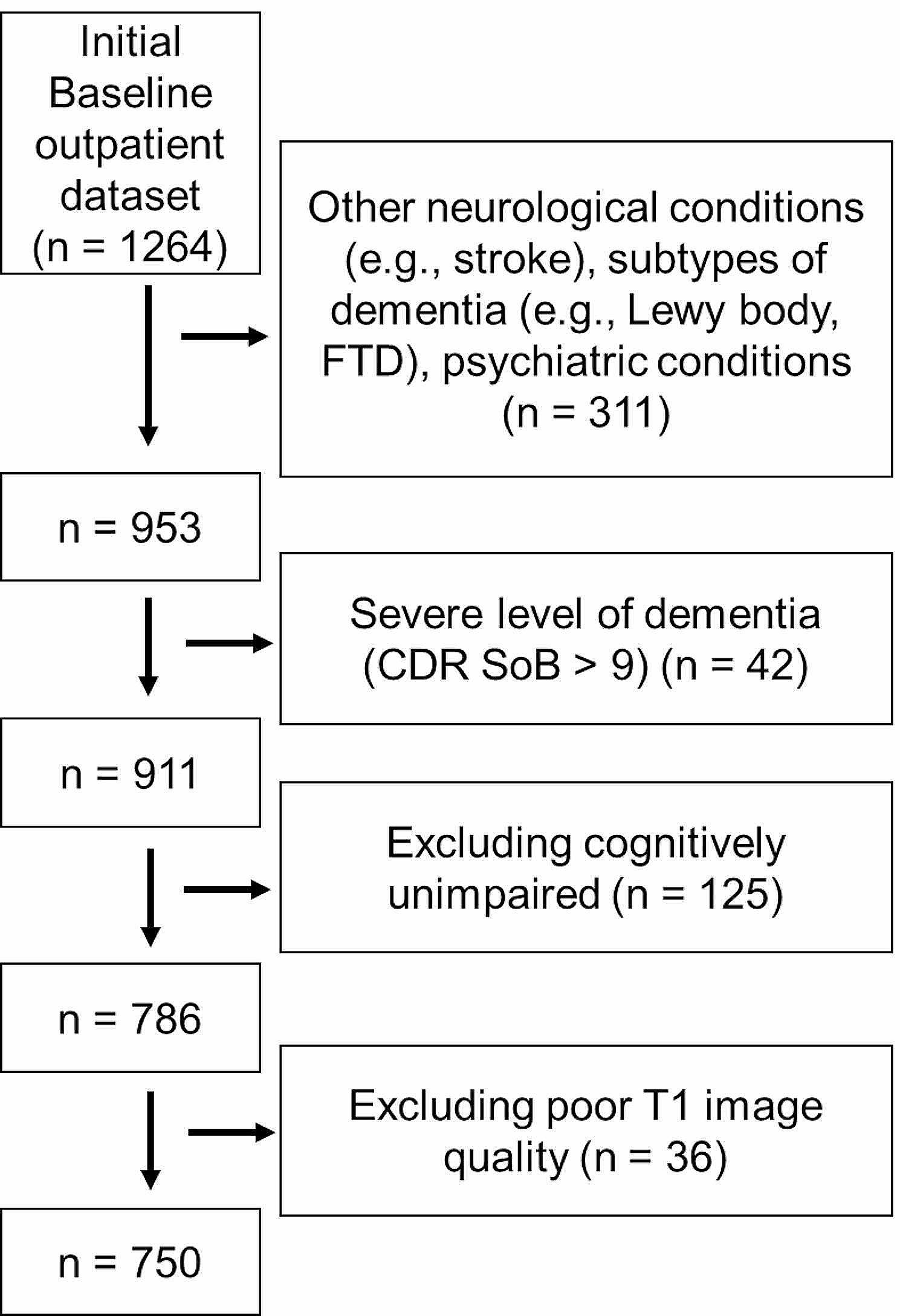
Exclusion flow diagram of the participants

The older adults were retrospectively recruited from outpatient database of SMG-SNU Boramae Medical Center for Dementia from January 2012 to December 2020. The participants underwent both neuropsychological assessment of dementia and a Magnetic Resonance Imaging (MRI) scan. This study was conducted under the Declaration of Helsinki, and the protocol was approved by the Institutional Review Board of SMG-SNU Boramae Medical Center (No.10-2020-295). The informed consent was fully waived by the Ethics Committee of SMG-SNU Boramae Medical Center due to the retrospective nature of the study and the analysis used anonymous clinical data. The current study included older adults with Mild Cognitive Impairment and Alzheimer’s disease. All participants received the Korean version of the Consortium to Establish a Registry for Alzheimer’s Disease neuropsychological battery (CERAD-K) [22] which provided normative decisions regarding the presence of cognitive impairment. The clinical diagnosis of MCI and dementia of AD was based on the core clinical criteria of National Institute on Aging-Alzheimer’s Association workgroups (NIA-AA) guidelines [23, 24]. Subjects suspected or diagnosed with dementia types including vascular dementia, Lewy body dementia, and frontotemporal lobe dementia were excluded. Those identified or suspected with significant neurological conditions including stroke, traumatic brain injury, meningioma, hemorrhage, normal pressure hydrocephalus, delirium, intellectual disabilities, schizophrenia, and bipolar disorders were excluded. Those with poor image quality due to head motion, and artifacts were also excluded based on preprocessing index. The exclusion flow is presented in Fig. 1.

Finally, a total of 750 older adults who met the screening criterion were analyzed, with a mean age of 75.9 years (SD = 7.4, range: 49–97), mean education of 8.0 years (SD = 5.0, range: 0–23), a higher sex ratio of females (61.9%) (Table 1). The participants with MCI had a larger proportion in the total dataset (AD, *n* = 358; MCI, *n* = 392). We confined our analysis within the severity staging of ‘mild’ impairment level considering the feasibility of the neuropsychological test (Clinical Dementia Rating sum of box score ≤ 9; Mean = 3.1, SD = 1.9).

### Delusion symptoms

Neuropsychiatric Inventory (NPI) was used to assess informant and clinician rating of delusion symptoms. NPI assesses the presence and severity of multiple neuropsychiatric symptoms of behavior and socioemotional regulation [25, 26]. The NPI was based on the semi-structured interview administered to the patients’ informants or caregivers, if available, and rated by clinical psychologists. The available informants were all assessed in the interview and the different qualities of the informant were fully considered by clinicians. Delusion item consisted of questionnaires assessing the presence of false beliefs that typically include themes of persecution (e.g., others are trying to harm or abandon), theft (e.g., others are stealing one’s property), infidelity (e.g., spouse is having an affair), and misidentification (e.g., not living in one’s own house; strangers are residing in one’s home). The item was rated from 0 to 3 scores across severity levels (0: No symptom, 1: Symptoms cause mild distress, 2: Symptoms are intractable and cause distress, 3: Symptoms are present with major distress).

In order to confirm the association with brain structure coinciding with a higher-order factor, an identical analysis was conducted with a sum score that covers the main behavioral symptom items (i.e., agitation/aggression, disinhibition, irritability). The current dataset showed a moderate correlation between the sum of behavioral symptom items and a delusion item (*r* = 0.43, *p* < 0.001). Furthermore, the correlation between the hallucination item and brain measures was additionally examined to infer the associating characteristic of delusion symptoms.

### Cognitive function

Cognitive functions were measured with the Korean version of the Consortium to Establish a Registry for Alzheimer’s Disease neuropsychological battery (CERAD-K) [22]. In the CERAD-K, the MMSE-KC (Korean version of Mini-mental status examination in the Korean version of CERAD Assessment Packet) is included as a battery, which required no additional license [27]. The CERAD-K battery measures multiple domains of cognitive function and facilitates the diagnosis of MCI and dementia. The battery contains the following subtests: Verbal fluency (the number of correct animal words generated in 60 s), Boston Naming Test (correctly named words when confronted with picture drawings), Word List Recall (immediate, delayed), Word List Recognition (subtraction of the number of false positives from the number of true positives), and Constructional Praxis (copy, reproduction), and Trail Making Test (TMT; total time spent to complete connect numbers or letters sequentially). TMT A / B measured the total time spent to complete the tasks. The test administration had set the maximum time limit at 360 s (TMT-A) and 300 s (TMT-B) based on administration instruction in CERAD-K [28]. The score was interpolated as the maximum time limit (360s or 300s) in the cases when the TMT was aborted or not feasible due to the following reasons: exceeded the time limit, unable to understand the rule, or committed more than five errors.

Five of the cognitive measures included the domain-wise sum of subtest scores. The scores were set to have zero means and divided with standard deviations. Each mediating domain of cognition and its composition subtests included language (Boston naming test, Animal fluency), episodic memory (immediate recall, delayed recall, recognition of word list learning), executive/speed (TMT A and B; inverse of log-transformation), visuospatial (constructional praxis copy and reproduction) and general cognition (MMSE-KC).

### Neuroimaging analysis

The neuroimaging data were collected in the MR scanner (3 Tesla, Achieva, Philips Medical Systems, Best, The Netherlands) to acquire a high-resolution T1 anatomical brain image with a 3D T1-weighted turbo field echo sequence (TR: 9.3 ms, TE: 4.6 ms, flip angle: 8˚, voxel size: 1.0 × 1.0 × 1.0 mm, slice thickness: 1 mm, 180 slices, image matrix: 224 × 224). We used a fully automated preprocessing procedure implemented in CAT12 r1450 (Computational Anatomy Toolbox; Structural Brain Mapping Group, Departments of Psychiatry and Neurology, Jena University Hospital, http://dbm.neuro.uni-jena.de/cat/) to apply a standardized analysis pipeline. All analyses and inspections were conducted by the main author (S.K). First, a spatial-adaptive non-local means denoising filter was employed [29]. Before image preprocessing, a weighted imaging quality rating (IQR) – an index that takes image spatial resolution, noise, and intensity bias into account – was automatically computed by CAT12 for each participant. The images with poor preprocessing performance were excluded (IQR > 2.3). Partial volume estimation was used to create a more accurate segmentation for the mixed tissue classes. Segmentation algorithms based on the adaptive maximum a posterior technique, implemented in CAT12, were used to classify brain tissue into gray matter, white matter, cerebrospinal fluid, and white matter hypointensities. All segmented GM images were visually checked for artifacts and motion effects.

For anatomical precision, surface-based morphometry by projection-based estimation of cortical thickness was conducted in the segmented images [30], which showed a comparable accuracy with other surface-based tools [31]. The values were extracted from the CAT12 region of interest (ROI) analysis pipeline. The regional definition of cortical thickness was based on Destrieux’s automatic parcellation of gyri and sulci resulting which provides 148 regional thickness measures [32]. For functional network labeling, gray matter density measures were extracted under modulated and spatially normalized segmentation GM images. All brain measures were residualized with the effect of sex and total intracranial volume to adjust for the nuisance effect. The resulting residuals were rendered uncorrelated with the covariates.

### Principal component analysis

Principal Component Analysis (PCA) with varimax rotation was conducted to summarize the distinctive multivariate dimensions of cortical thickness. Varimax rotation was applied after PCA in ways that maximize the sum of variances. The resulting output items were loaded onto the specified number of components. Varimax-rotation helps avoid PCA solution that typically results in components with intermixed loadings which leads to difficulty in interpreting the multivariate patterns. The resulting output is enhanced for a cleaner interpretation of the loadings while leaving an orthogonal basis of component scores.

**Fig. 2:**
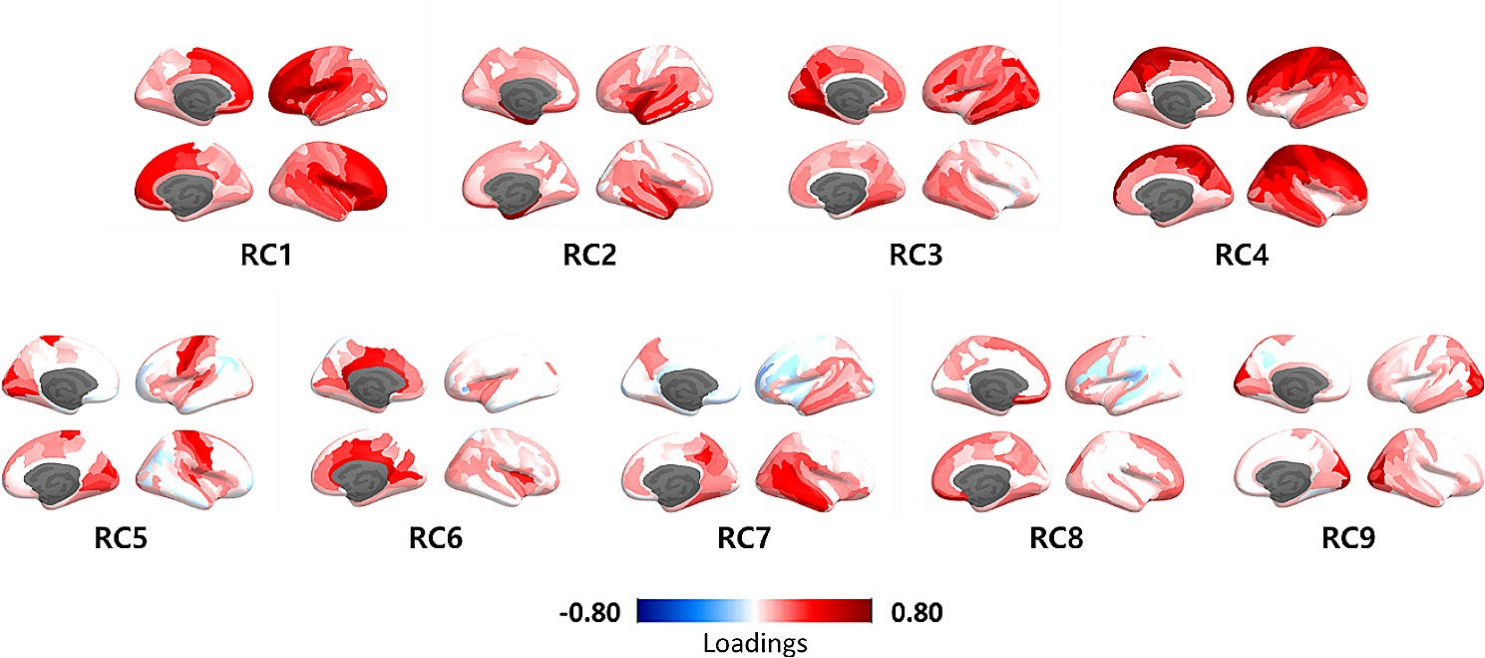
Principal component loadings after varimax rotation. The colored gradient indicates Standardized loadings (pattern matrix) based upon the correlation matrix

The number of rotated components was determined with parallel analysis. This analysis compares the scree of eigenvalues of the observed data with that of a random data matrix of the same size as the original. The random data matrix was generated with 50 iterations. Components with higher eigenvalues than the randomly generated data were considered meaningful units of the principal components. This parallel analysis was conducted using the *psych* package [33]. PCA with varimax rotation on the cortical thickness measures identified nine Rotated Components (RCs) (Fig. 2) which explained a total of 56% variance of brain measures.

To provide functional interpretability of RCs, the association between component scores and regional gray matter density measures that were based on functional network parcellation was additionally provided. A cortical parcellation map assigns 400 regions to the 17 functional networks [34]. It has been suggested that the covarying pattern of gray matter morphology partly indicates the large-scale influence of neurodevelopment and neuropathological changes [35, 36]. Based on the correlation between RC score and regional gray matter density measures, we labeled RCs with the highest correlating functional network regions.

### Statistical analysis

Partial correlation (Spearman’s method) between the NPI-Delusion rating and RC scores of cortical thickness was examined while controlling for sex and years of education. A total of nine RCs of cortical thickness were examined. Since the age and clinical diagnosis overlap with the main effect of interest, age-adjusted testing results were additionally provided.

To examine the overlapping basis of general cognition and delusion symptom, we explored the extent to which cognitive function mediate the relationship between cortical thickness pattern and delusional symptoms. The mediation effects on five of the mediation terms were explored to compare the specific contribution of cognitive domains separately. Based on the identified RCs that showed a significant association with delusion, further mediation analysis was conducted. The strength of the mediation effect was tested with standard errors estimated at 1,000 bootstraps. The effect of education and sex was controlled on every mediating term and outcome variable term (delusion). The total mediation effects were primarily tested and additionally examined the specific domain effects.

The principal component regression method lumps multiple correlating features into large components, thus we additionally explored regionally confined effects of interest. We examined whether ROI-level analysis corresponded to the preceding multivariable findings. After identifying regions that show rank-correlation with delusion symptoms across 148 regions of Destrieux’s parcellation (*p* < 0.01, uncorrected). In the ROI-level mediation analysis, regional mediation analysis assessed the relative strength of mediating effect within each ROI. The mediation strength was indicated by estimated mediation effect divided by the standard error (Z-values). All of the statistical analysis was conducted under R (4.2.1).

The *freesurfer_statsurf_display* MATLAB function was used to visualize the PCA loadings and ROI-level analysis results on cortical surface areas (Murdoch Children’s Research Institute Developmental, Imaging Group, 2017, https://chrisadamsonmcri.github.io/freesurfer_statsurf_display).

## Results

Based on the parallel analysis result, nine specified components were rotated. We identified distinct cortical thickness patterns by principal component analysis with varimax rotation (Fig. 2). The higher RC scores indicated a higher expression of the distributed regional thickness by their loading pattern. The correlations between component scores (RCs) were set to near zero. When examining the association with gray matter densities extracted with functional network parcellation, some thickness component patterns represented primary sensory cortical regions while the other components consisted of heteromodal associative cortical regions (Table 2).

We then examined the rank-order Spearman’s correlation between RC scores and NPI-delusion item rating. The three components (RC1, 2, 5) showed significant association with delusion under a lenient statistical threshold (*p* < 0.05, uncorrected; sex and education controlled) (Table 3). When the p-values were adjusted for multiple comparisons (FDR) or adding a covariate of age, only RC2 remained significant. When the same analysis was conducted with the NPI score that summed main behavioral symptom items (agitation, disinhibition, irritability), the RC2 showed consistent association pattern (temporal-insula; rho = -0.106, *p* = 0.004), while RC1 (prefrontal; rho = -0.036, *p* = 0.331) or RC5 (somatosensory and visual; rho = -0.045, *p* = 0.223) showed non-significant association. For additional clarification, the correlations with the hallucination item were further examined. It has shown that RC5 and RC1 was correlated with hallucination item (RC1: rho = -0.14, *p* < 0.001; RC5: rho = -0.07, *p* = 0.049) but RC2 did not (rho = -0.05, *p* = 0.169).

**Fig. 3:**
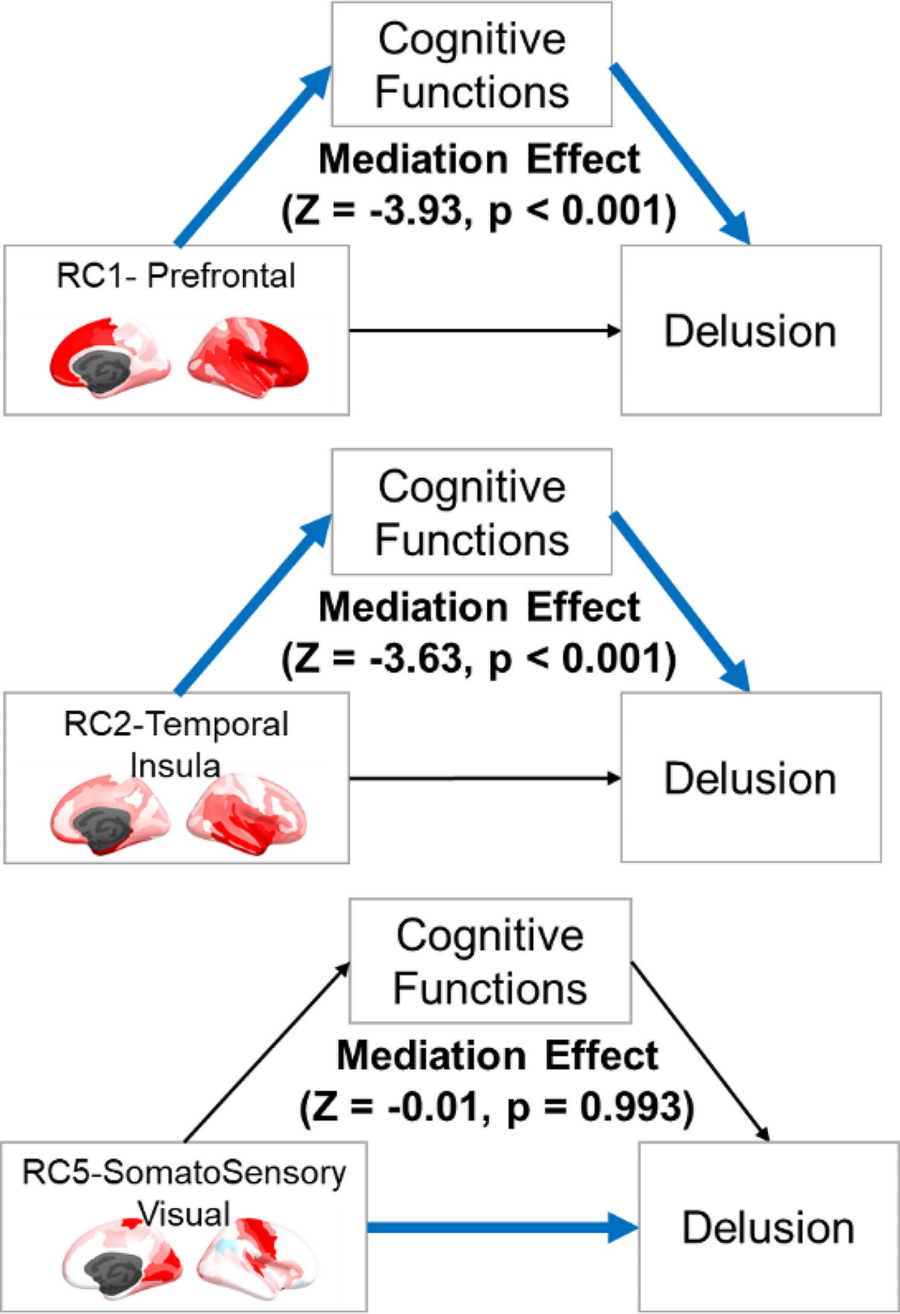
Mediation of cognitive functions between cortical thickness patterns (RCs) and delusion. The total mediation effect summed the five of the domain’s effects. Z-values indicate the estimated mediation effect divided by the standard error

The main brain components of interest (RC 1, 2, 5) which showed significant association with NPI-delusion were further explored with cognitive mediation analysis. The results showed that the associations of RC1 (prefrontal) and RC2 (temporal-insula) were significantly mediated by cognitive functions contrary to RC5 (somatosensory and visual) (Fig. 3). RC1 (indirect/total ratio = 0.46) and RC2 (indirect/total ratio = 0.59) showed a statistically significant cognition-mediation effect (*P* < 0.001), while RC5 showed no mediating effect (*p* = 0.993).

**Fig. 4:**
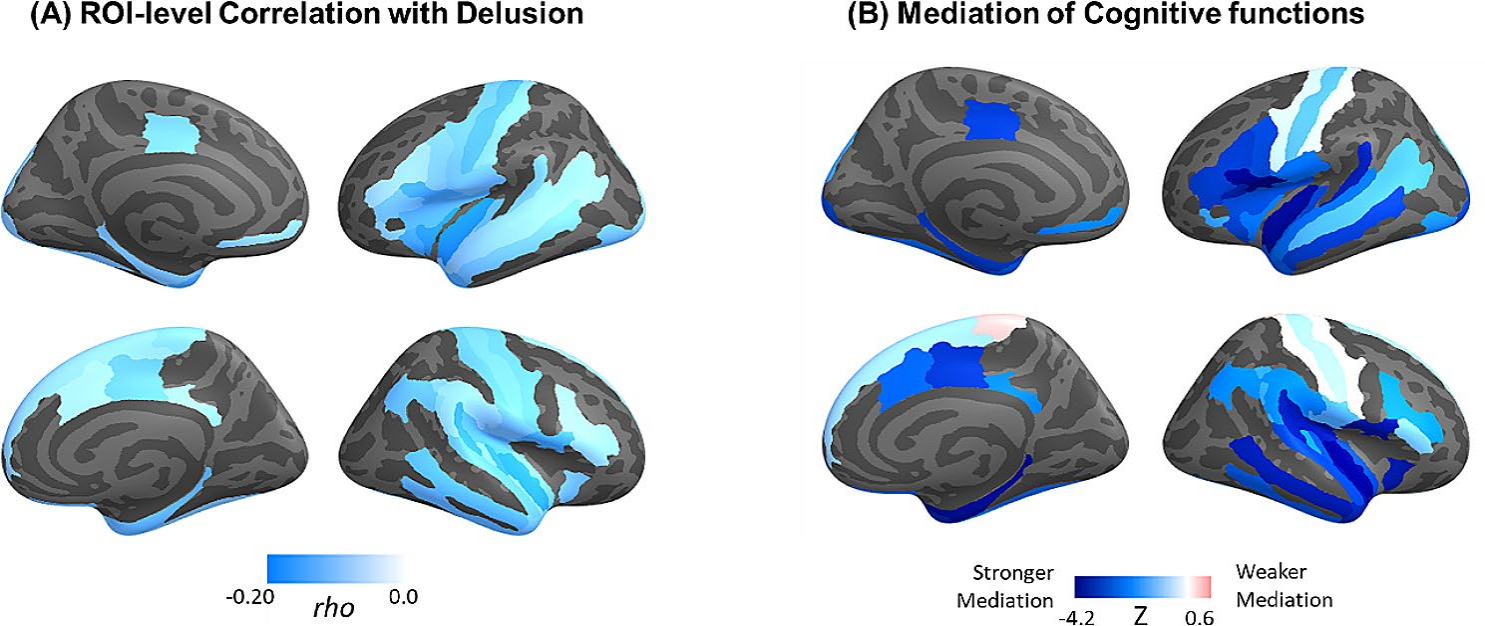
(**A**) ROI-level correlation (Spearman’s rho) between delusion symptom and cortical thickness (*p* < 0.01, uncorrected). (**B**) Cognition-Mediation effect (Z) within the thresholded correlates of delusion symptom. The Z-values indicate the estimated mediation effect divided by the standard error

The correlational and mediating effects were additionally confirmed by ROI-level analysis. When brain regions were masked with a relatively lenient threshold (*p* < 0.01, uncorrected), a stronger mediation effect was observed in regions of inferior prefrontal (opercular, triangular, orbital), temporal planum, lateral orbitofrontal, anterior and inferior insula, parahippocampal gyrus, and middle temporal cortex (Fig. 4). On the contrary, cortical thickness around the central sulcus showed minimal mediation of cognitive functions.

Lastly, the specified mediation effect of each cognitive domain was examined (Table 4). Among the total indirect mediating effects of RC1 and RC2, the executive/speed domain score significantly mediated the relationship between RCs and delusion. Although the relationship between RCs and episodic memory or MMSE-KC showed a robust association with the cortical thickness (X-M relationships), the associations did not sufficiently account for delusion (M-Y relationships).

## Discussion

The current study explored cortical thickness correlates of delusion among older adults with MCI or AD. We identified three distinct multivariable cortical thickness patterns that were negatively correlated with delusional symptoms. Among the cortical thickness patterns, those expressed as superior temporal and insular (RC2) or rostral, lateral, and orbital prefrontal (RC1) patterns were associated with delusions through mediating cognitive functions. On the contrary, a lower cortical thickness pattern expressed in the somatosensory and visual cortex (RC5) was associated with delusion not through cognitive function. These findings suggest distinct neurodegenerative and cognitive functional bases of delusion in dementing illness.

One notable observation in the current study was that subregions of the insular cortex were assigned to different orthogonal components of cortical thickness measures. While previous examinations have frequently reported insula as neural correlates of delusion, the findings were limited in their coarse and broad functional definition. The current study was able to specify the distinct units of thickness pattern within the insula. Our main finding showed that a lower cortical thickness pattern covering from superior temporal, anterior temporal pole, and ventral insular regions (RC2) showed a robust association with delusion in a cognitive impairment spectrum of Alzheimer’s disease, and the association remained consistent even after adding age covariate or multiple comparison adjustments. Numerous lines of literature indicate the distinct functions of insular subregions and their association with neurodegenerative disease [37, 38]. In a more extended scope, the component distributions surrounding the ventral insula highly resemble the map of the amygdala network especially of the aversion sub-network [39]. One previous study has also highlighted that cortical atrophy in the ventral insula within the aversion network exhibited more severe inappropriate trusting/approach behavior [40]. Unlike RC1 which extended to the dorsal part of the insula, RC2 covered the more ventral part of the insula, which is involved in processing gustation and visceral component of emotion [41]. This cortical pattern corresponded with our component labeling which showed that RC2 was strongly correlated with gray matter density measures of the limbic functional network [34]. The limbic network regions, labeled in the functional atlas along with the default mode network, are highly transmodal and topologically distal to regions taking charge of sensory inputs.

The functional role of temporal-insular regions may provide further insight into how Alzheimer’s disease patients develop themes of paranoia. Unlike typical symptoms of psychosis among young adults, which are exhibited as confusion in self to external boundaries and misattribution of mental experience to an external source, delusion among Alzheimer’s disease spectrum is associated with a more self-protective and hypervigilant state under loss of cognitive functioning [42]. Though speculative, the high prevalence of theft delusion, for example, may primarily result from failure to regulate psychological distance to external cues, which leads to appraising neutral stimuli as harmful and threatening. Moreover, the current study also has shown that RC2 correlation extends to other behavioral symptoms including agitation, irritability, and disinhibition. This suggests that RC2 is a neural basis not specific to delusion, and delusional ideation can interplay with other externalizing affective symptoms as a whole [7, 43, 44]. Possibly due to failures in processing visceral inputs, dysregulated interoception may lead to loss of adaptive negative feelings that motivate to comply with norm enforcement [45].

The widespread prefrontal cortical thickness pattern (RC2) also showed an association with delusion. The hypofrontality framework has well accounted for how attentional, inhibitory, and goal-directed functions can endanger reality monitoring [46, 47]. The finding specifically showed that more inferior frontal and lateral orbital regions seem to drive the deleterious effect of the hypofrontality. The included regions coincide with the previous studies that have highlighted ventrolateral frontal, orbitofrontal, superior frontal as key regions of delusions [8, 48, 49]. The component pattern also extends to the dorsal part of the insula which has shown consistent results in delusions of psychiatric conditions [50]. The subregional pattern implies that prefrontal correlates of delusions consisted of both ‘cold’ reasoning process and ‘hot’ affective processing and that previous hypofrontality frameworks of NPS may have overshadowed the importance of the socioaffective role of the prefrontal structure.

Another cortical thickness component identified as a delusion correlate was the pattern covering the somatosensory and visual cortex (RC5). Some studies have also reported that disruptions of the somatomotor and subcortical network were associated with common transdiagnostic psychopathology [51]. Also, a more posterior part of the insula and primary to secondary somatosensory cortex regions are involved in processing somatic pain and nociceptive regulation [52, 53]. It seems that the role of somatosensation in social perception or perceptual disorganization increases the burden of erroneous reasoning of delusion [54].

On the other hand, altered visual cortex function has also been observed in patients frequently exhibiting visual hallucinations [55]. RC5 also showed an association with hallucination item score, unlike RC2, supporting delusion mechanism due to regional atrophy involved in visual input and accompanying primary perceptual disorganization. Overall, a more perceptual base (RC5) of delusion may have distinct bases in the development of symptoms than affective integration (RC2) or controlled reasoning and appraisal (RC1).

When examining the mediating effect of multiple cognitive domains, only executive control and speed domain (Trail Making Test A and B) showed significant mediation. This suggests that even with a more progressed impairment in other domains including episodic memory (learning or delayed retrieval) or language function (proficient word generation or semantic knowledge), the risk of delusion may not be comorbid. Considering a large group difference between MCI and AD in cognitive functions and NPI severity, this lack of mediation effect suggests distinguished symptomatologies of delusion. Contrary to episodic memory or language tasks, executive control tasks explicitly assess cognitive processes that enable adaptive reasoning and integrate affective burden into judgment. The Trail Making Test requires a proficient grasp of stimuli-response contingencies and feedback-based learning from error trials. These cognitive correlates especially correspond with the lesion sites in the lateral inferior frontal, lateral orbital, and anterior insula cortex function [56]. Patients with relatively intact executive function may cope better when confronted with daily cognitive failures (e.g., ‘I lost my wallet and I need help to deal with my memory problem’) rather than augmenting erroneous reasoning into cognitive gaps (e.g., ‘I lost my wallet and somebody must have taken it’).

The overlap between brain regions involved in socioemotional versus general cognitive ability has been a controversial issue in its exact characteristics as distinct modules [57, 58]. In a previous study with lesion-symptom mapping, brain regional loss in the rostral and orbital frontal, insular, and temporal cortex showed a large corresponding overlap with impairment in both perceptual organization ability and emotional intelligence [59]. On the contrary, lesions in the precentral and postcentral cortex were more unique to impairment in emotional intelligence. This previous finding exquisitely matches the current study observations that lower thickness in somatomotor regions around the central sulcus correlated with more severe delusion symptoms but not through lower general cognitive functions.

We also highlighted some focal areas with explorative ROI-level analysis which warrant further discussions. When examining regions showing the strongest mediation of cognitive function, lateral orbital, inferior prefrontal cortex, and anterior insula showed a relatively high mediating effect of cognitive function. The lateral orbitofrontal cortex is involved in reversing one’s spontaneous reward representation and adapting to potentially punishing cues [60, 61]. The lateral part of the orbital cortex shows a strong functional connection with the inferior frontal cortex and insula [62], indicating its interplaying role in the process of goal maintenance and inhibitory control [63, 64]. The anterior insula, adjacent to the lateral orbital and inferior frontal cortex, bridges various distinct network modules, and the localized lesion in this area leads to more destructive behavioral impairment possibly due to its topological hubness [65, 66]. The heteromodal functional roles of these regions account for the shared neural bases of social, emotional, and cognitive abilities [17]. The current study provides further evidence that delusion, among other behavioral symptoms of dementia (BPSD), is characterized by impairment in one’s capacity to integrate behavioral, cognitive, and affective information to flexibly adapt to diverse social contexts and demands [16].

Lastly, the current study showed that structural abnormalities in the middle temporal, superior temporal, and parahippocampal cortex showed strong mediation of cognitive functions in explaining delusion. In previous studies, it has been suggested that thought disorder, known as a distinct but closely correlated with psychotic symptoms, is associated with structural abnormalities in the temporal cortex [67]. While the middle temporal gyrus processes primary semantic information received from the superior temporal cortex, but lack of further interplay between the middle temporal and inferior frontal cortex, which comprises the ventral pathway of language perception, can lead to grammatically and logically structured semantics [68]. The current study also consistently showed that domain-general control abilities accounted for delusion, rather than language domain scores that reflect a mere loss of semantic knowledge or fast phonology production.

We note several limitations in the current study. One of the major limitations of the current study was the lack of gray matter structure in subcortical regions. Since the current surface-based morphometrics were considered different from density-based measures of subcortical measures, only the former measures were used in our study. But future studies can integrate more valid morphometrics of subcortical structures (texture or volumetric) and examine more fine-grained underpinnings of delusion. Previous studies have suggested the importance of frontostriatal circuitry in the development of neuropsychiatric symptoms, and the occurrence of subtle ischemic attacks or pathophysiology may have affected cortical structures [69, 70]. Cortical thickness only provides indirect evidence of long-term neurodegenerative effects. Secondly, the cognitive abilities assessed with formal neuropsychological test batteries may have lacked sufficient coverage of brain functionality. For instance, tasks that comprise cognitive control ability covers lesion pattern of a small proportion of orbitofrontal regions whereas some other task (e.g., Iowa Gambling Task) may assess a more direct function of ‘hot cognition’ [56]. Adding cognitive tests that comprise ecologically relevant features of learning and decision-making would extend more coverage of NPS and delusion symptoms. Lastly, the current study failed to specify the exact type of psychosis. Although delusion symptoms share common erroneous cognitive processes, the pathophysiological bases may profoundly differ across various subtypes of delusion and future study needs to examine the distinguished mechanisms of delusion [71]. Overall, our findings suggest that multiple distinct subsets of brain regions underlie the delusions among older adults with cognitive impairment. Moreover, a neural loss may affect the occurrence of delusion in dementia largely due to impaired general cognitive abilities.

## Conclusions

We provided evidence that in the assessment of NPS and delusional symptoms in cognitively impairing populations, more extensive measurements of neurocognitive abilities can delineate even socioemotional functioning. While currently many of the primary care centers rely on the information from informants, future assessment tools can be more refined to assess frontotemporal and executive/speed deficits.

**Table 1:** Descriptive statistics by diagnoses of cognitive impairment

	Mild Cognitive Impairment	Alzheimer’s disease
n	392	358
Age	73.6 (7.0)	78.4 (7.1)
MMSE-KC	23.8 (3.5)	18.7 (4.5)
NPI Behavioral Symptoms ^*^	0.74 (1.44)	1.53 (1.99)
NPI Delusion (none/mild/moderate/severe)		
(%)	92.9/1.0/3.3/2.8	70.9/11.2/13.1/4.7
(n)	364/4/13/11	254/40/47/17

MMSE-KC: Mini-mental status examination Korean version CERAD packet; NPI: Neuropsychiatric Inventory

^*^ Sum of Neuropsychiatric Inventory (NPI) items of agitation, disinhibition, and irritability

**Table 2:** Anatomical and functional labeling of rotated components (RCs). (A) Cortical thickness regions with top-ranked rotated RC loadings. (b) RC correspondence to Schaefer’s functional network based on the top-ranked correlation between each RC and regional gray matter densities

	(A) Anatomical Regions	(B) Functional Network
RC1	Inferior frontal (Opercular, Triangular, Orbital), Dorsal insula, Middle frontal, Frontal pole, Lateral Orbital	Control, Ventral Attention
RC2	Temporal pole, parahippocampal, ventral insula, superior temporal	Limbic
RC3	Middle temporal, parieto-occipital, anterior occipital, calcarine, Inferior temporal	Dorsal Attenttion, Default
RC4	Superior parietal, Superior frontal, Precentral Postcentral, Precuneous	Somatomotor, Dorsal Attention
RC5	Central sulcus, paracentral, precentral, cuneous, superior temporal, occipital-temporal	Somatomotor, Visual
RC6	Posterior cingulate, middle cingulate,	Default, Dorsal Attention
RC7	Temporal (inferior, superior, middle)	Default
RC8	Medial orbitofrontal, ventromedial prefrontal (subcallosal)	Control, Default
RC9	Occipital (middle, superior), cuneous	Visual

**Table 3:** Partial correlation (Spearman’s Rho) between cortical thickness pattern scores (RCs) and delusion

	rho	p-value
RC1	-0.099	0.007
RC2	-0.163	< 10^− 5 a,b^
RC3	0.048	0.192
RC4	-0.048	0.194
RC5	-0.080	0.029
RC6	-0.032	0.377
RC7	-0.011	0.770
RC8	0.050	0.172

^a^ FDR-corrected *p* < 0.05. ^b^*p* < 0.05 with age covariate added

**Table 4:** Mediation effects of cognitive functions by domains

	RC1 (Prefrontal)			RC2 (Temporal-insula)			RC5 (Somatosensory / visual)		
	B(SE)	Z	p-value	B(SE)	Z	p-value	B(SE)	Z	p-value
Mediators									
Language	0.003(0.004)	0.733	0.464	0.015(0.014)	1.042	0.298	0.002(0.003)	0.738	0.460
Memory	-0.003(0.003)	-1.050	0.294	-0.019(0.013)	-1.477	0.14	0.002(0.003)	0.592	0.554
Visuospatial	0.000(0.003)	0.128	0.898	0.001(0.008)	0.170	0.865	0.000(0.001)	0.064	0.949
Executive/Speed	-0.017(0.006)	-2.694	0.007	-0.024(0.008)	-2.909	0.004	-0.004(0.004)	-0.965	0.334
MMSE-KC	-0.009(0.005)	-1.703	0.089	-0.027(0.014)	-1.952	0.051	0.003(0.004)	0.682	0.496
Indirect effect (X-M-Y)	-0.026(0.008)	-3.339	0.001	-0.054(0.014)	-3.805	< 0.001	0.003(0.008)	0.413	0.680
Total Effect (X-Y)	-0.066(0.026)	-2.514	0.012	-0.084(0.027)	-3.098	0.002	-0.078(0.029)	-2.666	0.008

## Data Availability

The data, which support this study, are not publicly available but may be provided upon reasonable request to the corresponding author. Dataset can be accessed as either full unprocessed raw database or final analyses set.
